# Distinct electrophysiological correlates of low- and high-level visual consciousness with minimized post-perceptual processing

**DOI:** 10.1016/j.isci.2026.117853

**Published:** 2026-10-15

**Authors:** Annika Hense, Antje Peters, Torge Dellert, Maximilian Bruchmann, Thomas Straube

**Affiliations:** 1 Institute of Medical Psychology and Systems Neuroscience, University of Muenster, Münster, Germany; 2 Otto Creutzfeldt Center for Cognitive and Behavioral Neuroscience, University of Muenster, Münster, Germany; 3 Goethe University Frankfurt, Epilepsy Center Frankfurt Rhine-Main, Department of Neurology, University Hospital Frankfurt, Frankfurt, Germany

**Keywords:** neural correlates of consciousness, EEG/ERP, visual awareness, levels of processing

## Abstract

Conscious visual phenomenology comprises multiple representational levels, from basic features to integrated percepts, raising the question of how the neural correlates of consciousness (NCC) differentiate accordingly. Event-related potential (ERP) studies identified early occipitotemporal negativities as reliable NCC; yet, it remains unclear whether these capture conscious content beyond low-level features and extend to higher-order representations. Moreover, whether later ERP components index higher-level conscious percepts independent of task-related processing remains contested. The present study addresses this question using an inattentional blindness (IB) paradigm that isolates three representational levels of conscious perception (no, low- or high-level awareness) for an identical stimulus under minimized post-perceptual processing. Across 85 healthy participants, low-level line perception was indexed by an enhanced early occipital negativity, whereas high-level face perception additionally recruited a right-lateralized occipitotemporal negativity, followed by medial posterior positivities. These findings reveal systematic spatiotemporal differentiation of awareness-related potentials across representational levels of consciousness under task-irrelevant conditions.

## Highlights

• Awareness negativities vary in timing and topography across levels of awareness
• Focal occipital negativity was associated with low-level awareness of lines
• Occipitotemporal negativity was associated with conscious face representation
• Additional parietal positivities was associated with high-level face representation

## Graphical abstract

**Figure:**
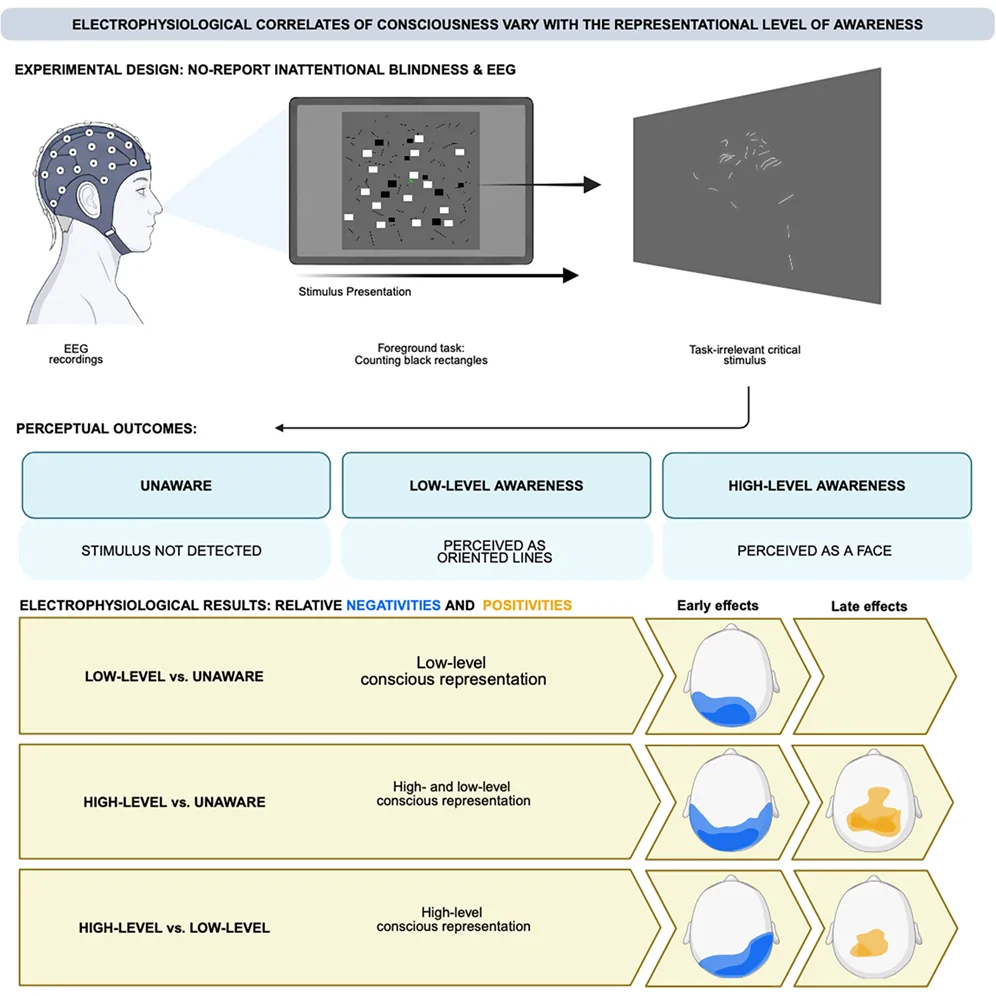

## Teaser

Systems neuroscience; Sensory neuroscience; Cognitive neuroscience

## Introduction

Conscious perception enables individuals to navigate complex environments by integrating sensory inputs into coherent, meaningful experiences.^1^ Yet, the underlying neural mechanisms remain poorly understood, with competing theories and methodological challenges.^2^^,^^3^^,^^4^

Event-related potential (ERP) studies on visual neural correlates of consciousness (NCC) have identified the early visual awareness negativity (VAN), differentiating seen from unseen stimuli at occipitotemporal sites approximately 200 ms post-stimulus.^2^^,^^5^^,^^6^ The VAN aligns with theories proposing posterior sensory regions as sufficient for visual awareness,^3^^,^^7^ such as recurrent processing theory (RPT)^8^ and integrated information theory (IIT).^9^ Alternative approaches, such as the global neuronal workspace theory (GNWT),^10^^,^^11^ posit that conscious experience depends on the global broadcasting of information to cognitive systems, associated with a frontoparietal “ignition.” However, the P3b, commonly linked to this process,^12^ appears more reliably correlated with post-perceptual and task-related processes^13^^,^^14^ rather than consciousness itself.^15^^,^^16^^,^^17^^,^^18^^,^^19^^,^^20^^,^^21^^,^^22^ Nevertheless, selective involvement of frontoparietal regions, as well as varying electrophysiological potentials, have been observed under certain task-irrelevant experimental conditions.^16^^,^^18^^,^^23^^,^^24^^,^^25^ A comprehensive framework that reconciles these inconsistent observations remains lacking.

The lack of a unified framework is assumed to partly reflect the inherently complex and multifaceted nature of consciousness,^1^^,^^26^^,^^27^ involving variation in awareness-related neural signatures across different representational levels of conscious processing.^28^^,^^29^^,^^30^^,^^31^^,^^32^ Notably, we use the terms *consciousness* and *awareness* interchangeably throughout this work to denote phenomenal awareness, i.e., the experiential, subjective character of a conscious percept.^3^^,^^33^ The content of this experience, what is represented in awareness, can be instantiated at different levels of the visual processing hierarchy, which we refer to as the *representational level of conscious content*. These representational levels are organized within the hierarchical ventral occipitotemporal cortex,^34^^,^^35^^,^^36^^,^^37^ where neurons encode progressively complex sensory features, from elementary visual attributes to abstract representations, enabling robust object identification across diverse sensory inputs.^38^^,^^39^ Recent perspectives suggest that consciousness operates at different thresholds for low-level perceptual features and high-level abstract representations,^28^^,^^31^^,^^40^^,^^41^^,^^42^ with divergent findings attributed to heterogeneous operationalizations of visual awareness,^43^ ranging from simple grating detection^44^ to complex cognitive judgments.^45^ Existing studies may probe different facets of consciousness, eliciting distinct processing depths and neural mechanisms.^27^^,^^28^^,^^29^^,^^31^ Indeed, visual consciousness likely emerges through dynamic, multilayered neural processes with content-dependent spatiotemporal dynamics that transcend reduction to a singular, static electrophysiological signature.^4^^,^^19^^,^^30^^,^^37^

Prior ERP studies aiming to differentiate visual representational levels have linked the VAN to low- and the P3b to high-level feature awareness.^29^^,^^31^^,^^46^^,^^47^^,^^48^^,^^49^ However, these studies have typically relied on indirect operationalizations, either through varying attentional and cognitive demands^29^^,^^46^^,^^47^^,^^48^ or through trial-wise subjective clarity ratings to differentiate different consciousness thresholds.^48^^,^^49^^,^^50^ Both approaches risk confounding proper NCC with pre- and post-perceptual processes. Observed ERP modulations consequently likely capture mixtures of differential attentional and task-related processes.^35^ Attentional manipulations, stimulus types, and task requirements have been shown to modulate the VAN,^17^^,^^51^^,^^52^ with other studies showing no such effects.^18^^,^^53^^,^^54^ Furthermore, the proposed VAN selectivity for low-level conscious contents is challenged by evidence showing that a VAN can also be elicited under high-level operationalizations.^17^^,^^50^ Moreover, spatiotemporal variations of awareness-related ERPs have been insufficiently addressed in previous investigations.^29^^,^^46^^,^^47^^,^^48^ Taken together, prior approaches may have lacked both the methodological precision to dissociate representational levels of conscious content from identical input and the analytical sensitivity to detect systematic spatiotemporal variation of awareness-related ERPs, underscoring the need for an approach that allows to capture representational levels of conscious experience under task-irrelevant conditions.

The present study aimed to investigate how awareness-related ERPs are modulated by low versus high levels of conscious representations arising from identical visual input while minimizing confounding influences from task-related or post-perceptual activity. We developed a novel inattentional blindness (IB) paradigm that employs both a low- and high-level awareness contrast (see Figure 1). By systematically varying participants’ prior knowledge about a task-irrelevant abstract face stimulus composed of lines (uninformed, informed about low-level features, or informed about high-level features), the design yielded to unawareness of the stimulus, low-level awareness of the constituent lines, or high-level awareness of the lines as a face, while holding stimulation and task demands constant across groups. This approach sheds light on how different awareness-related ERP components are modulated by different representational levels of conscious content, contributing to understanding the neural mechanisms underlying the multifaceted phenomenal nature of visual awareness.

**Figure 1:**
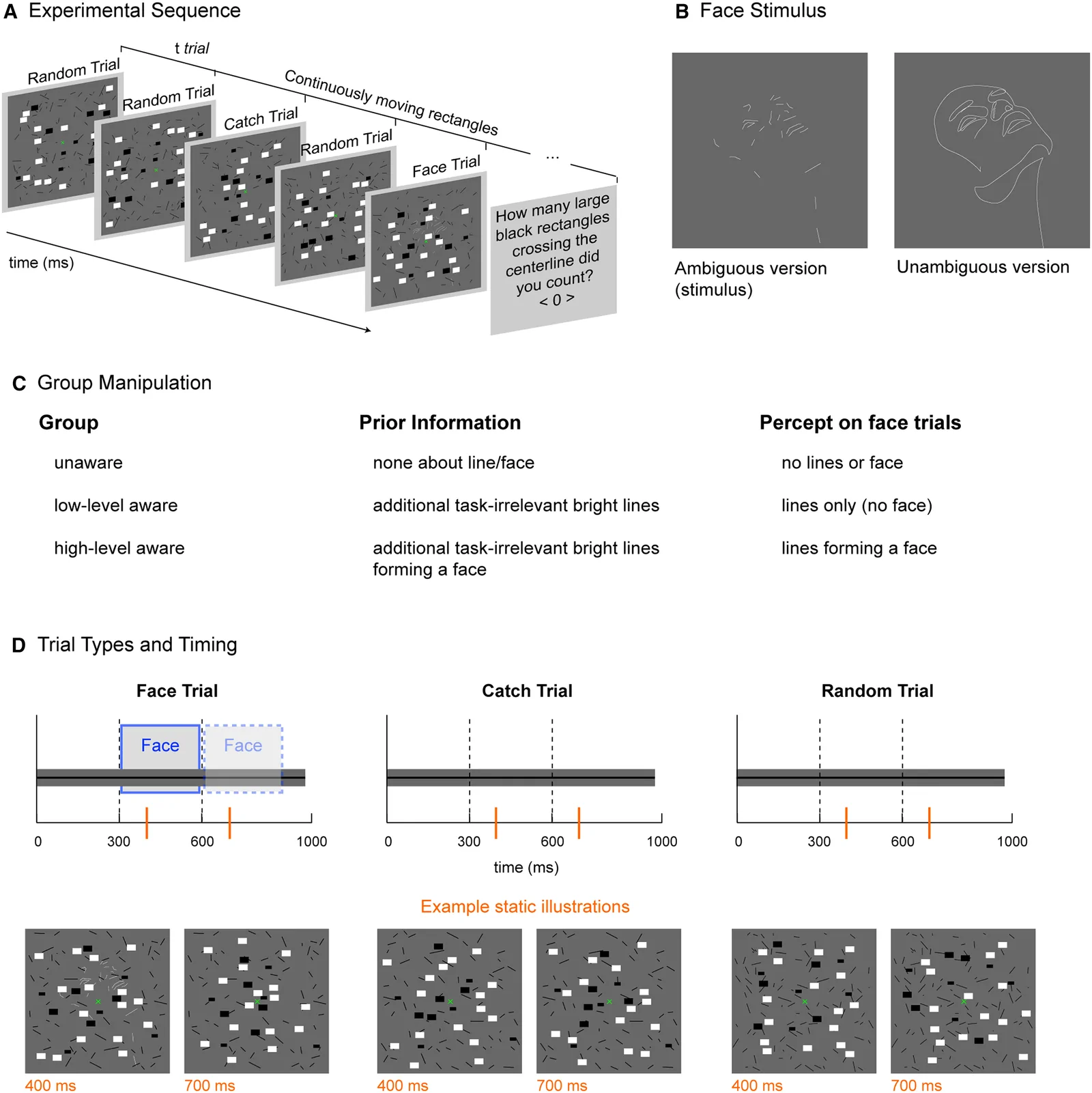
Experimental paradigm and stimuli (A) Example trial sequence. Participants performed a visual distractor task in which black and white rectangles continuously moved within a quadratic frame. While maintaining central fixation on a green cross, they counted the number of large black rectangles crossing the imaginary horizontal midline within each block. Line configurations in the background defined the trial types (random, catch, and face trials) and were entirely task-irrelevant, with a duration of t_*trial*_ = 1,000 ms. (B) Face stimulus. Left: ambiguous version used as the experimental stimulus; right: corresponding unambiguous line drawing. (C) Group manipulation. Participants were assigned to one of three prior-information conditions with expected perceptual outcomes on face trials. This yielded three groups (unaware *n* = 32, low-level aware *n* = 23, high-level aware *n* = 30) under identical task demands and physical stimulation for final analysis; participants whose perception did not match the expected outcome were excluded. (D) Trial types and timing. Top: in face trials (left), 70 black lines were presented throughout trial duration. An additional 32 bright lines constituting the face (marked by blue frame) appeared for 300 ms at either 300 or 600 ms post-trial onset (here: 300 ms post-trial onset). Catch trials (middle) were identical to face trials (70 black lines at trial onset) but omitted the face stimulus. ERPs were time-locked to face onsets and matched time points in catch trials, with catch trials serving as a baseline for enabling differential neural responses (face-catch). Random trials (right) contained 102 black lines presented throughout trial duration. Orange vertical lines mark the time points (400 ms and 700 ms) at which example visual displays are illustrated below. Bottom: Example stimulus snapshots at two time points post-trial onset across trial types. Black line configurations remain constant throughout the trial duration. In face trials, the face stimulus added 32 bright lines to the background display for 300 ms (contrast enhanced here for visualization). Foreground rectangles moved continuously throughout all trials.

## Results

### Behavioral data

#### Task performance

Individual task performance was quantified as the median across normalized relative deviations between participants’ counts and actual crossings across experimental blocks, with lower values indicating higher accuracy (Figure 2B). Participants demonstrated high overall accuracy, with a mean deviation (averaged across participants’ individual median deviations) of *M* = 0.11 (*SD* = 0.05), representing an overall average deviation of 11%. Welch’s ANOVA revealed significant group differences in median deviations (*F*[2,50] = 5.22, *p* = 0.009, *BF*_*M*_ = 17.33, *ω*^*2*^ = 0.14), with post hoc analyses showing the unaware group exhibited lower median deviations (*M* = 0.09, *SD* = 0.04) than the high-level group (*M* = 0.13, *SD* = 0.06; *t* = −3.25, *p* = 0.006, *BF*_*10*_ = 20.4, *d* = −0.83). The low-level group’s performance (*M* = 0.1, *SD* = 0.04) did not significantly differ from either the unaware (*t* = −1.01, *p* = 0.578, *BF*_*01*_ = 2.5, *d* = −0.28) or the high-level group (*t* = −2.09, *p* = 0.102, *BF*_*01*_ = 0.71, *d* = −0.57).

**Figure 2:**
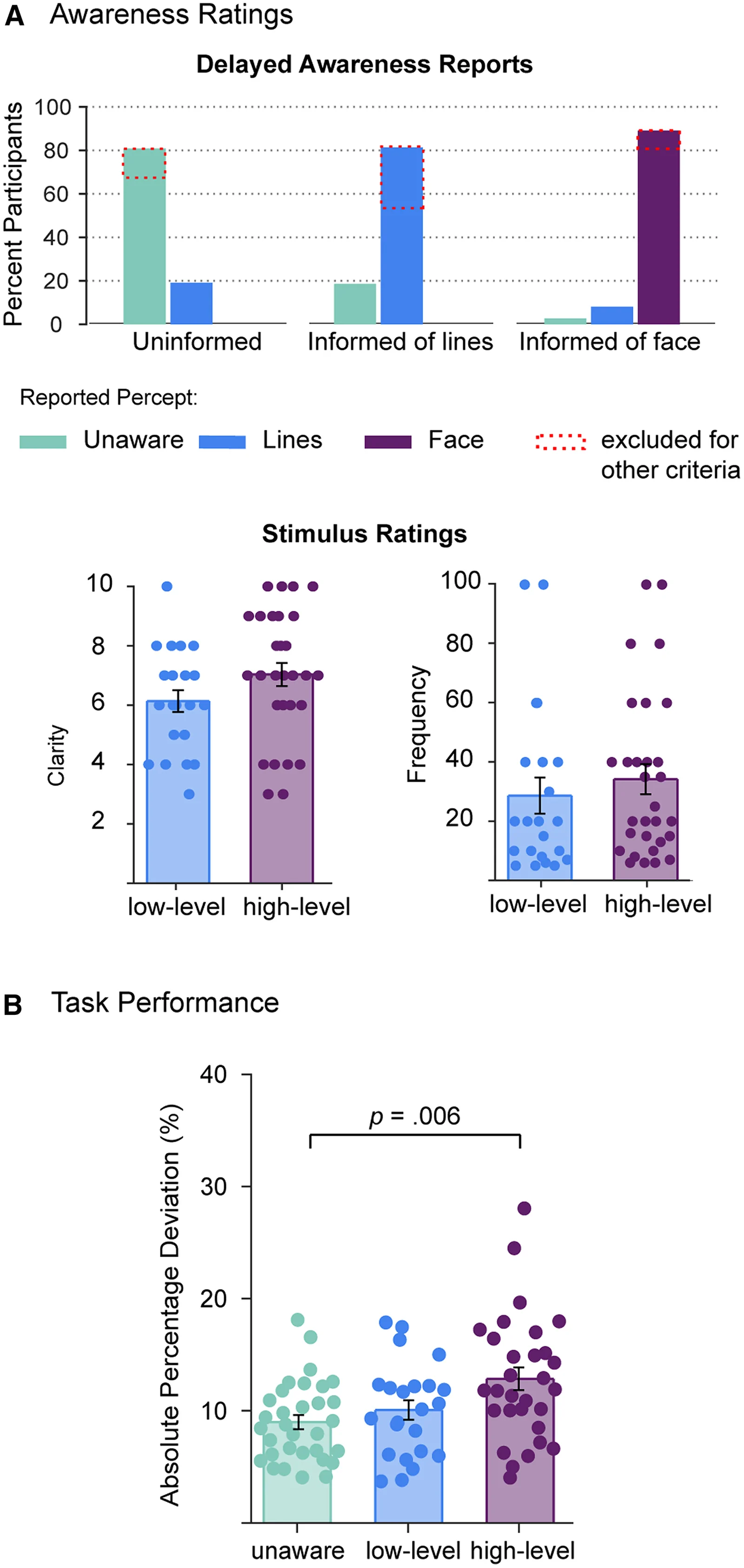
Behavioral data (A) Top, delayed awareness reports: Analysis included only participants whose reported perception matched their assigned experimental condition. Bar plots show the percentage of participants reporting each perceptual outcome relative to the total groupwise recruited sample within each experimental group (recruited: 43 uninformed, 43 low-level, 37 high-level). Bottom, stimulus ratings: post-experimental subjective ratings from the final low- and high-level aware groups regarding the critical stimulus. Subjective clarity ratings (1–10 scale) revealed no significant between-group differences. Frequency ratings represent participants’ estimates of how many times they perceived the stimulus throughout the experiment. Block-wise estimates were summed to generate total exposure counts for the bar plot. Range estimates (e.g., “20–30 times”) were rounded to the higher value (e.g., 30 times). Black horizontal lines represent group means with error bars indicating the standard error of the mean (SEM). (B) Task performance in the visual counting task across experimental conditions. Each dot represents an individual participant’s performance, displayed as the median across blocks of block-wise absolute percent deviation between participants’ reported counts and actual crossings. Black horizontal lines represent group means with error bars indicating the SEM. Lower absolute percent deviations signify more accurate counting performance, with zero indicating perfect accuracy. The p value shown in the figure refers to a two-tailed Games-Howell post hoc test following Welch's ANOVA.

#### Awareness ratings

Awareness ratings confirmed successful experimental manipulation, with more than 80% of recruited participants reporting the intended perceptual outcome (Figure 2A top). Retrospective clarity ratings revealed no significant differences between the low-level (*M* = 6.14, *SD* = 1.73) and high-level (*M* = 7.03, *SD* = 2.13) group (*t*[49] = −1.678, *p* = 0.10), indicating that the manipulation targeted representational levels rather than subjective clarity differences.

### EEG data

The EEG analysis using TFCE revealed distinct spatial and temporal patterns across the awareness contrasts (see Figure 3). The low-level versus unaware contrast showed a significant negative difference, driven by an effect in posterior occipital electrodes, spanning 190–315 ms post-stimulus (see Figure 4A). This effect showed a clear posterior occipital focus, most pronounced at electrode Oz (*t*_*max*_ = −3.98, *p*_TFCE_ = 0.012) and adjacent posterior occipital electrodes O2, O1, Iz, and PO3 at 291 ms post-stimulus. The high-level versus unaware contrast revealed a significant negative difference, driven by an effect with an extended duration (194–350 ms post-stimulus; see Figure 4B) and a broad bilateral occipitotemporal distribution, encompassing lateral temporal (TP7/8), parietal (P6/7/8/9/10), and occipital (Iz, Oz, O1/2, POz, PO4, PO7/8) sites. The effect peaked at electrode Iz (*t*_*max*_ = −5.31, *p*_TFCE_ < 0.001) at 244 ms post-stimulus and was most pronounced in occipital electrodes and adjacent occipitoparietal sites. The high-level versus low-level contrast yielded a later negative cluster spanning from 233 to 350 ms post-stimulus (see Figure 4C), which was driven by an effect in right-lateralized occipitotemporal electrodes, maximal at electrode P10 at 334 ms (*t*_*max*_ = -4,42, *p*_TFCE_ = 0.001) and including the neighboring right-lateralized channels TP8, P6, P8, PO8, and O2.

**Figure 3:**
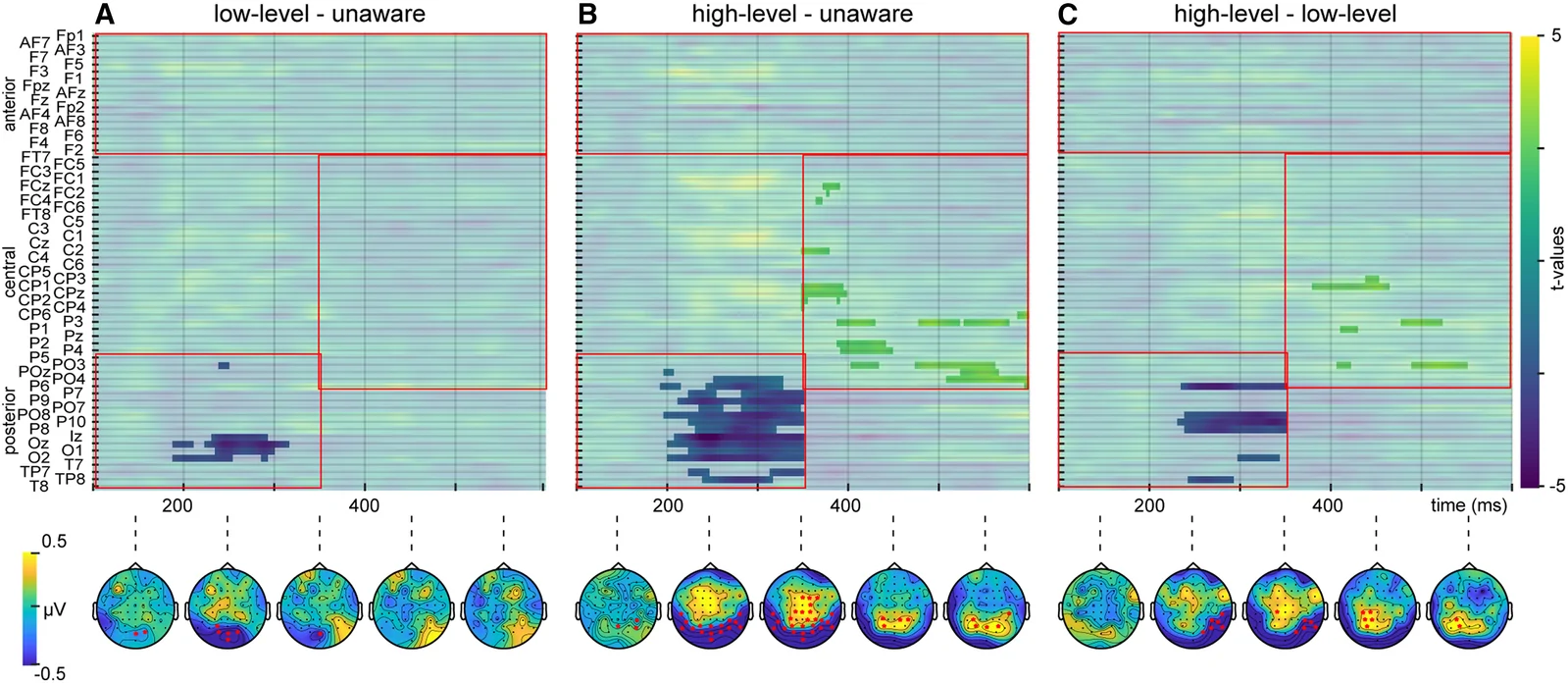
Results from the mass univariate analysis of EEG data for all three awareness contrasts and across all statistical comparisons (A–C) The maps show significant differences in stimulus-specific responses (face - catch trials) for each awareness contrast. Each row on the *y* axis represents an individual electrode, with time plotted in milliseconds on the *x* axis from 100 to 600 ms post-stimulus onset. Significant *t* values (*p* < 0.05) after TFCE correction are displayed at full opacity, while non-significant *t* values are shown in 50% transparency. The color intensity represents the magnitude of the *t* values. Red rectangles denote electrodes and time windows subjected to statistical analysis: posterior electrodes tested for negative clusters between 100 and 350 ms, and frontocentroparietal electrodes tested for positive effects from 350 to 600 ms (see EEG data analysis). Topographical voltage distributions (in μV) for each awareness contrast are displayed below in corresponding 100 ms intervals from 100 to 600 ms post-stimulus onset. Vertical dashed lines connect each topographic map to its corresponding time interval in the univariate analysis plots above. Channels that are significant for at least one sample during the depicted interval are highlighted by a red asterisk.

**Figure 4:**
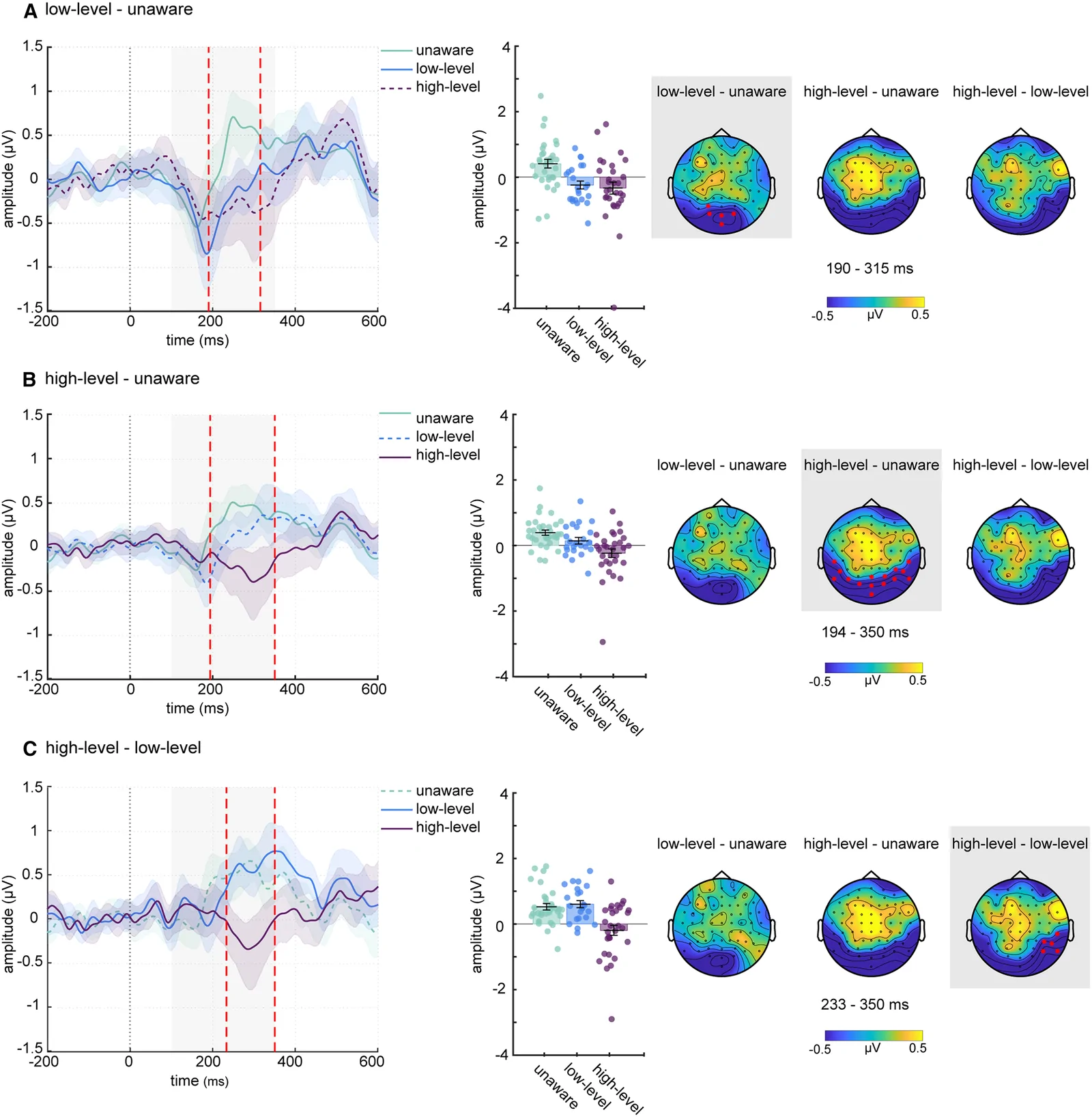
Negative ERP differences in posterior electrodes for the three awareness contrasts Significant negative differences in differential (face-catch) ERP responses found in the three awareness contrasts analyzed across posterior electrodes within a 100–350 ms time window: low-level vs. unaware (A), high-level vs. unaware (B), and high-level vs. low-level (C). Left: averaged ERPs for each group across cluster electrodes, with shaded areas depicting 95% bootstrap confidence intervals. Light gray rectangles indicate the time window of interest (100–350 ms), and red dashed lines mark the temporal extent of the significant cluster found for the respective awareness contrast. For each awareness contrast, the ERP waveforms of the groups statistically compared are shown with solid lines, while the third condition not included in that particular contrast is displayed with a dashed line. Middle: scatterplots showing individual participants’ ERP amplitudes (μV) for differential responses (face-catch), averaged across the cluster electrodes and cluster time, separated for each group. Colored bars with black horizontal lines and error bars represent groups’ means and SEMs. Right: Topographical voltage distributions for each awareness contrast, averaged over the cluster time (indicated below each map) found for the analyzed awareness contrast indicated by light gray rectangles. Voltage differences are shown in microvolts (μV) and red asterisks mark significant cluster electrodes.

Two positive clusters emerged in the high-level versus unaware contrast: an earlier broad centro-parietal cluster around 350–448 ms, maximal at CP1 at 362 ms (*t*_max_ = 2.99, *p*_TFCE_ = 0.041), followed by a cluster driven by an effect in posterior parietal electrodes spanning 475–600 ms, that peaked in electrode P3 at 491 ms (*t*_max_ = 3.31, *p*_TFCE_ = 0.032; see Figure 5A). Accordingly, two similar positive clusters were found for the high-level versus low-level contrast. A centro-parietal positive cluster spanning 381–463 ms post-stimulus and maximal in electrode CP1 at 451 ms (*t*_max_ = 3.08, *p*_TFCE_ <0.046) followed by a small posterior parietal cluster in P3 and PO3 around 479–549 ms, peaking in electrode PO3 at 518 ms (*t*_max_ = 3.17, *p*_TFCE_ <0.046; see Figure 5B). Exploratory analyses revealed no significant P3b-related awareness effects in electrode Pz (*F*[2,82] = 1.210, *p* = 0.303). Furthermore, no significant central negativities between 200 and 600 ms were found in any of the three awareness contrasts.

**Figure 5:**
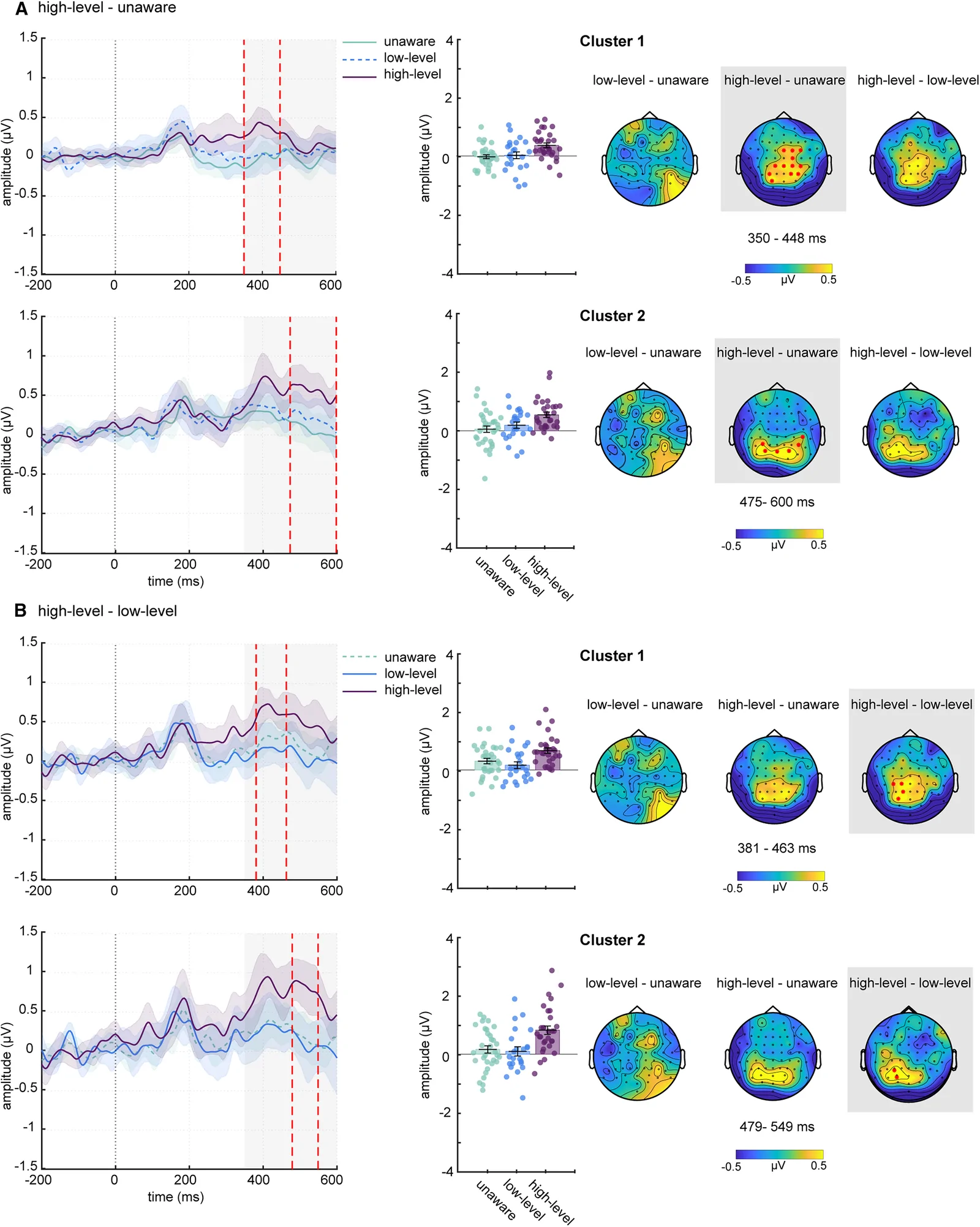
Positive ERP differences in frontocentroparietal electrodes for the three awareness contrasts Significant positive differences in differential (face-catch) ERP responses found in the three awareness contrasts analyzed across frontocentroparietal electrodes within a 350–600 ms time window: two clusters in the high-level vs. unaware contrast (A), and two clusters in the high-level vs. low-level contrast (B). Note that no significant positive effects were found for the low-level vs. unaware contrast. Left: averaged ERPs for each group across cluster electrodes, with shaded areas depicting 95% bootstrap confidence intervals. Light gray rectangles indicate the time window of interest (350–600 ms), and red dashed lines mark the temporal extent of the significant cluster found for the respective awareness contrast. For each awareness contrast, the ERP waveforms of the groups statistically compared are shown with solid lines, while the third condition, not included in that particular contrast, is displayed with a dashed line. Middle: Scatterplots showing individual participants’ ERP amplitudes (μV) for differential responses (face-catch), averaged across the cluster electrodes and cluster time, separated for each group. Black horizontal lines with error bars represent groups’ means and SEMs. Right: Topographical voltage distributions for each awareness contrast, averaged over the cluster time (indicated below each map) found for the analyzed awareness contrast indicated by light gray rectangles. Voltage differences are shown in microvolts (μV) and red asterisks mark significant cluster electrodes.

An additional exploratory TFCE analysis was performed without *a-priori* channel selection using two-sided comparisons per awareness contrast in the time range between 100 and 600 ms and is provided in the supplementary material (Figure S1).

To ensure that observed neural differences reflected awareness-related effects rather than attentional confounds, moderation analyses were conducted examining whether task performance moderated group differences in cluster-specific ERP amplitudes. Seven linear regression models were fitted to test for performance × group interactions across all identified clusters (3 negative, 4 positive clusters). All models demonstrated significant explanatory power (all *p* ≤ 0.006, adj. *R*^*2*^ ≥ 13.35%), indicating that the predictors accounted for substantial variance in ERP amplitudes. Crucially, none of the performance × group interaction terms reached statistical significance (for the dummy-coded groups corresponding to each cluster’s defining contrast: all *p* ≥ 0.264), showing that task performance did not moderate group differences in any cluster. Significant main effects of group persisted in the expected directions (for the dummy-coded groups corresponding to each cluster’s defining contrast: *p* ≤ 0.006), confirming that neural differences between awareness conditions remain statistically robust when task performance was held constant. These findings indicate that the identified neural signatures represent genuine awareness-related effects rather than attentional confounds (see supplement for complete statistical details). Additionally, we tested for correlations between task performance and average amplitude differences in each observed cluster to clarify the contribution of attentional engagement. No significant correlations were observed (all uncorrected *p* ≥ 0.146; see Figure S2 in supplemental information).

## Discussion

The present ERP study introduces an IB paradigm conceived to investigate how electrophysiological NCC are modulated across representational levels of conscious perception. This approach aims to minimize confounding influences of task-requirements, physical stimulation, and post-perceptual processing. Low-level awareness of lines elicited an enhanced early occipital negativity, while high-level face perception correlated with a spatiotemporally extended negative potential. Compared to low-level awareness, the high-level conscious perception specifically involved a later right-lateralized occipitotemporal negativity accompanied by positive components over parietal and medial posterior sites. The Figure 6 schematically summarizes this main pattern of findings. The neural signatures of consciousness thereby manifest in distinct spatiotemporal patterns that correspond to different representational levels of conscious perception, providing evidence for hierarchically organized conscious processing within the visual system.

**Figure 6:**
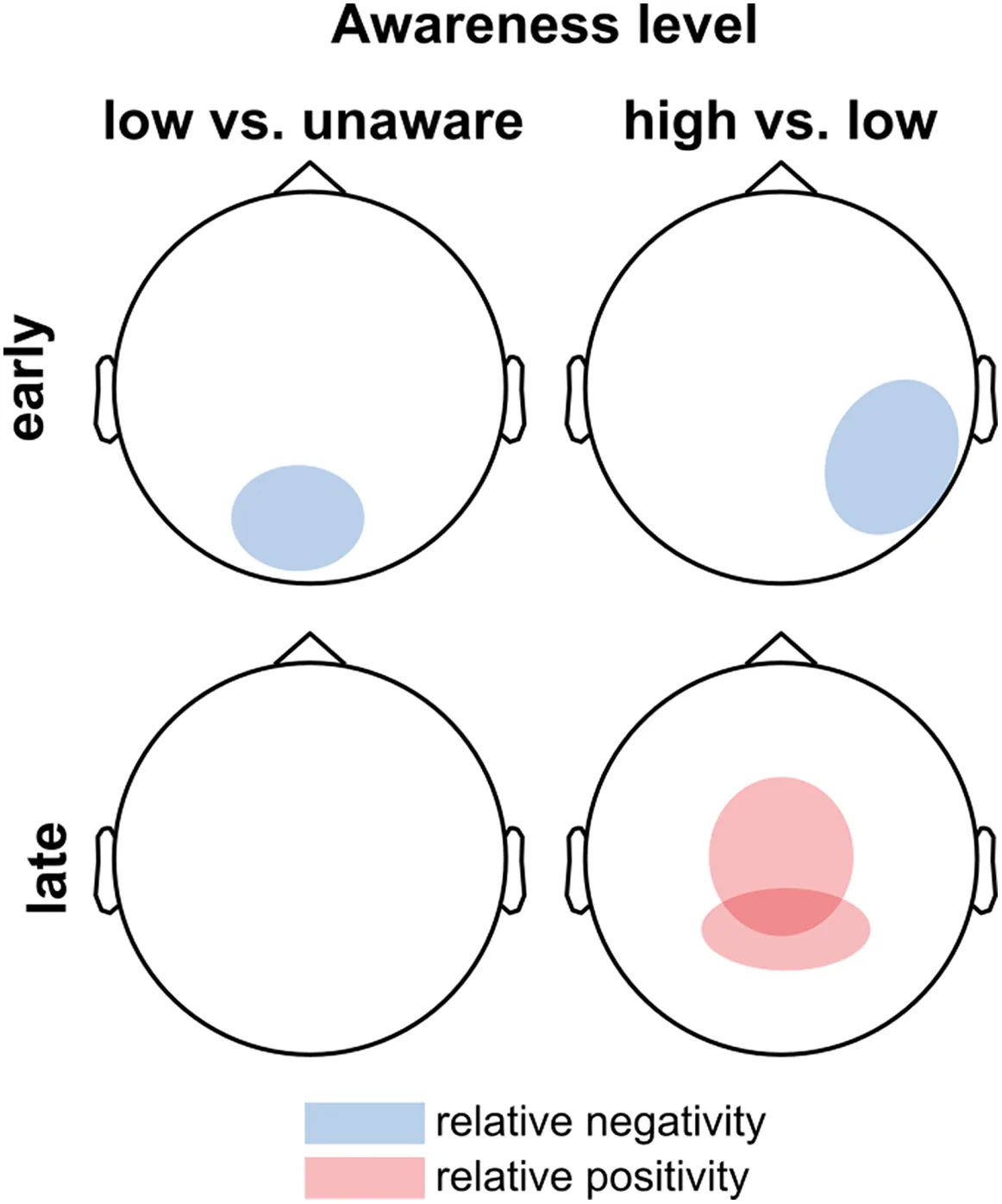
Summary of main ERP effects Schematic illustration summarizing the main ERP effects in the early (100–350 ms) and late (350–600 ms) time windows of interest. Relatively negative (blue) and positive (red) effects reflect comparisons of differential ERP responses (face-catch) between the low-level and unaware groups (left column) and between the high-level and low-level groups (right column) at the respective electrodes of interest.

The present findings confirm the role of awareness-related negative potentials over occipitotemporal sites as reliable NCC^5^^,^^6^^,^^55^ and suggest that their precise spatiotemporal patterns depend on the representational level of conscious experience. Low-level perceptual awareness of the face stimulus elicited a typical VAN-like potential in posterior occipital electrodes, corresponding to existing views suggesting that this component reflects the stimulus’ low-level visual features crossing the threshold into conscious awareness.^46^^,^^47^^,^^48^^,^^49^ In contrast, high-level conscious perception of the identical stimulus as an abstract face elicited a spatiotemporally extended negative potential compared to the low-level awareness-related negativity. Specifically, the high-level versus unaware contrast, encompassing conscious perception of both basic visual features and their integration into a conscious face, revealed an expanded scalp distribution across occipitotemporal sites. While this negativity emerged with a similar onset latency of around 190–195 ms (see Figures 4A and 4B), it exhibited prolonged temporal dynamics compared to the low-level specific component. These findings align with the more elaborate processing required for integrating visual elements into a coherent object representation across successive stages of the ventral visual stream.^56^^,^^57^^,^^58^ Crucially, isolating the neural signature specific to high-level face perception from low-level visual line detection revealed a distinct right-lateralized awareness-related negative potential peaking around 334 ms, which was absent in the low-level versus unaware contrast. These results are consistent with earlier findings of content-specific NCC for conscious face perception^17^^,^^22^ and a VAN for high-level conscious contents.^17^^,^^50^ This component’s right lateralization aligns with the right-hemispheric specialization for categorical facial feature analysis within the bilateral face processing network,^59^ further supported by evidence that asymmetric interhemispheric recruitment of the right occipital face area underlies frequently observed right-sided activation during face processing.^60^ The present study critically extends earlier findings by showing that awareness-related negativities vary systematically in topography and temporal dynamics as a function of the representational level of the conscious content, operationalized as basic visual feature detection versus abstract higher-level face perception. Accordingly, the electrophysiological neural signatures underlying conscious visual perception likely do not reflect a single neural phenomenon but are far more differentiated than previously assumed.^2^^,^^5^

This challenges the view that early negative potentials specifically index low-level conscious perception^29^^,^^40^^,^^46^^,^^47^^,^^48^^,^^49^ (but see Wiens et al.^50^) whereas high-level conscious perception is indexed by the task-related P3b.^29^^,^^47^^,^^48^ Instead, our findings support a more nuanced, dynamic model in which awareness-related negativities over posterior brain regions differentiate between the low- and high-level conscious percept, rather than indexing low-level perception alone. Most prior studies addressing levels of conscious processing relied on visibility ratings across task requirements emphasizing either low- or high-level stimulus features, inferring differential ERP sensitivity to representational levels indirectly from modulation patterns rather than directly isolating conscious percepts at specific levels.^29^^,^^40^^,^^46^^,^^47^ This approach conflates perceptual experiences with task-induced cognitive and attentional processes^13^^,^^14^ and is limited by the inherent confound between representational levels and degrees of awareness, reflecting perceptual qualities of precision, intensity, or confidence, which operate at all processing levels.^35^ Moreover, the precise distribution of visibility ratings and related ERP patterns is shaped by numerous factors, including stimulus properties, task type, and individual scale interpretations, additionally constraining interpretability of these studies.^28^^,^^35^^,^^36^ Other designs have employed visibility ratings to directly distinguish detection and identification thresholds, yielding mixed results.^48^^,^^49^^,^^50^ However, while reduced stimulus visibility can impair identification processes, such designs primarily inform debates concerning the graded versus discrete nature of consciousness, rather than elucidating the hierarchical organization of conscious perception.^35^ While VAN amplitudes systematically vary across visibility ratings,^61^^,^^62^ the specificity of this neural signature for distinguishing low-level versus high-level conscious contents remains unresolved. The methodological limitations and insufficient sensitivity to spatiotemporal variations in negative potentials may account for inconsistent results in previous studies.^29^^,^^40^^,^^46^^,^^47^^,^^48^^,^^49^^,^^50^ At the same time, the present findings indicating the VANs’ sensitivity to high-level conscious representations aligns with IB studies that identified enhanced occipitotemporal negative potentials when applying a higher-level operationalization of awareness based on perceptual integration.^17^^,^^22^^,^^62^^,^^63^

Beyond the occipitotemporal awareness-related negative potentials, we neither found a general central positivity resembling the P3b as proposed by the GNWT^10^^,^^11^^,^^64^ nor a central negativity as observed by some previous NCC studies minimizing task-related post-perceptual confounds in vision^16^^,^^18^ and audition.^65^^,^^66^ However, our findings additionally reveal varying late positive potentials associated with high-level conscious face perception that were absent during low-level conscious perception. Both the high-versus low-level and high-level versus unaware contrast demonstrated late positive effects which exhibited a biphasic profile, comprising an earlier centro-parietal component (∼350–450 ms) followed by a more spatially circumscribed posterior-parietal positivity (∼480–550 ms), though likely distinct from the P3b.^67^ The latter is traditionally maximal at electrode Pz. The observed positive potentials lacked both the sudden amplitude increase and Pz-centered focus typical of the P3b, as between-group comparisons revealed no statistically significant differences at Pz during the typical P3b time window. These findings align with existing evidence challenging brain-wide ignition and the P3b as reliable NCCs in the absence of task-related decision-making.^15^^,^^16^^,^^17^^,^^18^^,^^19^^,^^20^^,^^21^^,^^22^^,^^63^^,^^68^ Notably, previous no-report ERP studies employing high-level consciousness thresholds for simplified face line drawings similarly found no significant awareness-related P3b but weak, nonsignificant positive potentials at occipital sites.^17^^,^^22^ This suggests that the detection of these late positive effects may critically depend on the sensitivity of the analytical approach to detect weak ERP effects with atypical P3b topographies and time windows. Similar positive deflections emerged in both contrasts involving high-level perception (high-level vs. unaware and high-level vs. low-level). Since the high-level versus low-level contrast subtracts out neural activity associated with low-level conscious perception, the positive signatures likely reflect shared high-level conscious processing.

Given their rather posterior parietal topographical distribution, and occurrence under task-irrelevant conditions, these effects may be driven by parietal regions or interactions involving frontoparietal and occipito-temporal regions^16^^,^^23^ which have been reported to discriminate between seen and unseen stimuli under no-report conditions.^23^ However, the prefrontal cortex remains a particularly debated region regarding its involvement in conscious perception^7^^,^^17^^,^^23^^,^^69^^,^^70^^,^^71^^,^^72^^,^^73^ and the neural sources of these effects cannot be determined from the scalp EEG alone. Their occurrence in the absence of task-related decision-making and selective for the high-level conscious percept, with no comparable effects observed for conscious low-level perception, indicate a content- or level-dependent process.

One interpretation is that these positivities reflect recurrent top-down feedback supporting the stabilization and disambiguation of abstract visual representations through interactions between posterior visual and higher-order temporal-parietal cortices.^30^^,^^74^ High-level interpretations are more underdetermined by sensory input alone and may require additional top-down processes to resolve perceptual ambiguity, whereas low-level content depend more directly on bottom-up sensory features.^74^^,^^75^ Alternatively, the late positivities may reflect global cognitive accessibility, analogous to Sergent et al.’s “‘global playground,”^66^ in which information becomes available to higher-level cognitive processes without triggering full frontoparietal broadcasting. In the present study, these late positivities would be selectively recruited when consciously perceived content carries sufficient representational behavioral significance to engage frontoparietal networks, even in the absence of task demands. A further possibility, consistent with findings by Kronemer et al.^19^ is that the observed positivities reflect a later pulse of awareness-related signal amplification mediated by limbic and arousal networks, which were engaged more strongly by the face percept than by the lines, potentially reflecting greater affective and social salience of facial percepts. Given EEG’s limited ability to resolve underlying neural anatomical sources, future fMRI studies are needed to delineate the contribution of specific brain regions within the experimental framework established here. If future studies indicate parietal cortical sources, theoretical accounts might interpret this differently. Functionalist accounts might interpret such effects as parietal involvement associated with higher-order functions such as attentional selection,^76^^,^^77^^,^^78^ working memory,^79^ or decision-making.^80^^,^^81^ Others, such as proponents of the IIT or RPT might relate this to the neural substrate of conscious experience involving temporal-parieto-occipito cortical regions distinct from those involved in higher-level cognitive functions.^3^^,^^7^^,^^16^^,^^23^ The presence of these late positivities may depend on the relative engagement of these processes, influenced by cognitive demands of certain paradigms or awareness manipulations. Whether such higher-level functions can be clearly separated from conscious processing remains an open question.^19^^,^^66^^,^^82^

Our findings support a framework of consciousness that moves beyond the notion of a single neural correlate of consciousness, emphasizing instead the multifaceted nature of conscious experiences.^1^^,^^33^^,^^82^^,^^83^^,^^84^^,^^85^^,^^86^ High-level conscious experiences constitute an inherent aspect of phenomenal awareness with distinctive qualitative properties transcending the perception of their low-level constituent elements,^28^^,^^29^ distinct from access consciousness or post-perceptual processes.^14^^,^^37^ Previous NCC research has conflated this distinction with low and high-level awareness, mapping high-level experience to access consciousness and P3b-related post-perceptual activity, and low-level perception to phenomenal experience and the VAN.^46^^,^^47^^,^^48^ However, we suggest that although high-level invariant concepts are often more easily reportable than low-level experiences,^37^^,^^87^ their phenomenal character should be investigated under minimized post-perceptual processes.^14^ The present experimental design takes a first step toward this by eliciting low- and high-level conscious representations of an identical stimulus under task-irrelevant conditions, thereby minimizing confounds from attentional and cognitive processes.^13^^,^^14^

### Conclusions

Taken together, these findings show that visual awareness-related negativities exhibit a varying spatiotemporal profile as a function of the representational level of conscious experience, rather than being restricted to a uniform signature of low-level awareness, while medial centro-parietal positivities are only observed during high-level perception. Our data are consistent with theories that localize content-specific NCCs in feature-selective posterior regions across representational levels, although their neural generators need to be established in future work. The precise functional role of the late positive effects, and their potential dependency on salience and residual attentional engagement, remains to be determined, and theoretical frameworks may interpret these effects differently. Our study provided the first attempt to manipulate perceptual experience across three levels for the same visual input, while minimizing task-related confounds. While the results suggest a spatiotemporal differentiation of awareness-related potentials depending on representational levels of conscious perception, future work could, for example, use different visual objects, include within-participants control conditions and combine EEG and fMRI research.

### Limitations of the study

Nevertheless, the present findings should be interpreted in light of multiple limitations, which also point to directions for future research. Most of these limitations stem from awareness being operationalized via a prior-information manipulation combined with retrospective, delayed awareness reports. This approach enabled controlled perceptual conditions though it comes with some trade-offs outlined below.

A central limitation concerns the reliability of subjective retrospective reports. A main critique of the IB is that it measures what can be remembered and reported rather than what is perceived, reflecting inattentional amnesia. Yet, the IB has been shown to reflect a perceptual rather than a memory failure.^27^^,^^88^ Moreover, because the awareness assessment asked explicitly and specifically about the critical stimulus, it is unlikely that an experience of a salient face, or the sudden onset of unexpected lines, would have been forgotten or left unreported. Nevertheless, the more graded measures should be interpreted more cautiously, particularly frequency and clarity ratings.

Also inherent to this approach is that awareness of the critical stimulus is not assessed on a trial-by-trial basis, implying uncertainty about how consistently each condition reflected the intended representational level in individual trials. The “aware” conditions may include a heterogeneous mixture of trials in which the critical stimulus is fully experienced, partially experienced, or unseen. Among high-level aware participants, for instance, individual trials may have involved only low-level feature awareness, or no conscious experience of the stimulus. Critically, such trials would underestimate the high-level NCC, rendering our approach conservative. However, the study yielded robust VAN effects, components that are typically small,^2^^,^^5^^,^^17^ indicating that the delayed-report procedures were sufficiently sensitive to detect awareness-related ERPs. Notably, the reverse scenario, the low-level aware conditions comprising trials of high-level awareness, is unlikely given the salience and high memorability of faces.

Moreover, as groups were assigned *a priori* rather than determined by spontaneous noticing, the groups are less likely to differ systematically in susceptibility to IB^89^ than in previous designs.^17^^,^^22^ Nevertheless, excluding participants whose reports did not match their assigned conditions may still have biased the final sample to disproportionately representing aware participants who are less susceptible to IB. Future work could address this by applying the present logic to a within-subject design, e.g., using unknown task-relevance^18^ or other no-report approaches.^19^^,^^23^ Moreover, different figures could be used as critical stimuli, with participants being informed about one of them but not the other to further reduce the influence of interindividual variability and enable within-group comparisons.

Crucially, although control analyses showed that the ERP effects were not explained by group differences in task performance, consistent with them not being primarily driven by differences in attentional allocation to the task, the present IB paradigm does not entirely exclude non-perceptual contributions apart from explicit task relevance^90^ that may not manifest in task performance. In particular, the prior information manipulation may have introduced varying expectation and meaningfulness of the stimulus in the aware groups but not in the unaware group.

Moreover, the face stimulus in the high-level awareness condition not only carries high-level representational content but also social and behavioral significance, making it difficult to fully dissociate effects of the representational level from salience-related processes or stimulus-driven relevance. Both the prior information phase and the high-level face percepts in particular may have engaged stimulus-driven relevance processing^91^^,^^92^^,^^93^ or attentional capture^94^ not reflected in task performance measures. Critical next steps would require future studies to incorporate continuous eye-tracking to allow stronger conclusions regarding the contribution of attentional processes. Moreover, varying representational levels of conscious content across multiple non-facial categories and matched for salience would help to separate effects specific to representational levels from face-specific processing and implicit meaningfulness induced by the information phase. Combined EEG-fMRI would further allow to localize the neural sources of the observed ERP effects and help to adjudicate between accounts implicating broader temporal networks^66^ or arousal-related regions including the anterior cingulate cortex and anterior insula.^19^ Notably, previous no-report IB fMRI studies with abstract face stimuli from our lab showed mainly effects in the occipito-temporal cortex, in particular the fusiform gyrus.^17^^,^^95^ Additionally recording pupillometry would further help for evaluating arousal-related responses. Finally, manipulating stimulus ambiguity to probe the role of prior knowledge and top-down processes,^74^ as well as the transfer to different report and no-report designs^18^^,^^23^ would allow more precise evaluation of the contribution of different considered processes that may accompany high-level awareness without necessarily constituting NCC per se.

## Resource availability

### Lead contact

Requests for further information and resources should be directed to and will be fulfilled by the lead contact, Annika Hense (annika.hense@uni-muenster.de).

### Materials availability

This study did not generate new unique reagents.

### Data and code availability

• All data reported in this study will be shared by the lead contact.
• This study does not report original code.
• Any additional information required to reanalyze the data reported in this study is available from the lead contact upon request.

## Acknowledgments

The authors would like to thank Hannah, Lisann, and Ida for their assistance in data collection. We thank all the volunteers who participated in this study.

## Author contributions

Conceptualization, A.H.; data curation, A.H. and A.P.; formal analysis, A.H. and A.P.; investigation, A.H. and A.P.; methodology, A.H., A.P., M.B., T.D., and T.S.; project administration, A.H. and A.P.; software, A.H., A.P., M.B., and T.D.; validation, A.H., A.P., M.B., T.D., and T.S.; visualization, A.H. and A.P.; writing – original draft, A.H.; writing – review and editing, A.P., M.B., T.D., and T.S.; funding acquisition, T.S.; resources, T.S.; supervision, T.S.

## Declaration of interests

The authors declare no competing interests.

## STAR★Methods

### Key resources table

**Table:** 

REAGENT or RESOURCE	SOURCE	IDENTIFIER
**Software and algorithms**		

MATLAB R2019b	MathWorks	RRID:SCR_001622
BESA Research 6.0	BESA GmbH, Gräfelfing, Germany	RRID:SCR_009530
FieldTrip (version 20250106)	Oostenveld et al.^96^	RRID:SCR_004849
Psychophysics Toolbox (Psychtoolbox-3)	Kleiner et al.,^97^	RRID:SCR_002881
Threshold-free cluster enhancement (TFCE)	Mensen and Khatami^98^; Smith and Nichols^99^	Implemented in FieldTrip (RRID:SCR_004849)

**Other**		

Sequence data, analyses, and resources related to the ultra-deep sequencing of the AML31 tumor, relapse, and matched normal	This paper	http://aml31.genome.wustl.edu
Resource website for the AML31 publication	This paper	https://github.com/chrisamiller/aml31SuppSite

### Experimental model and study participant details

#### Subjects

A total of 127 volunteers were recruited at the University of Münster via public advertisements. All participants had normal or corrected-to-normal vision and no history of psychiatric or neurological disorders. Participants provided written informed consent and received monetary compensation, including a performance-dependent bonus. Only participants with sufficient behavioral and EEG data were included in the final sample.

In line with previous IB EEG studies,^17^^,^^21^^,^^22^ this study targeted 32 participants per experimental condition following the application of primary exclusion criteria (recruited: 47 uninformed, 43 low-level informed, 37 high-level informed): misalignment between reported stimulus perception and assigned experimental group (nine from the uninformed, eight from the low-level, and four in the high-level group; total 21), technical failures during EEG acquisition (four from the uninformed, three from the low-level, one from the high-level informed group, total 8), excessive EEG artifacts (one participant) and behavioral task performance outliers (one participant performed below 3 times the group’s interquartile range). To increase statistical power, additional participants were subsequently excluded from the sample if they reported an insufficient number of perceived stimuli (<5; nine from the low-level and two from the high-level group, total 11). The final sample for behavioral and EEG analyses consisted of 85 participants (61 female, 24 male; age 18–34 years, *M* = 23.01, *SD* = 3.03), comprising 32 in the unaware group (24 female, 8 male; age *M* = 23.06; *SD* = 3.07), 23 in the low-level group (13 female, 10 male; age *M* = 23.00, *SD* = 2.88), and 30 in the high-level group (24 female, 6 male; age *M* = 23.17, *SD* = 3.38). The groups did not differ in age (one-way ANOVA: *F*(2, 82) = 0.02, *p* = 0.981) or in gender distribution (*X*^2^(2) = 3.81, *p* = 0.149).

Age and gender were not the focus of the present study and were not entered as a factor into the main analyses. As the three groups did not differ significantly in age or gender distribution (see above), these variables are unlikely to account for the observed group differences, but their influence on the awareness-related effects were not specifically examined. Apart from age and gender, no further sociodemographic data were collected. The study was approved by the institutional ethics committee of the Medical Association of Westphalia-Lippe and the University of Münster (reference number 2019–049-f-S).

### Method details

#### Stimuli and presentation

The experimental design aimed at creating three experimental groups with respect to participants’ perception of a critical face stimulus: (1) an unaware group having no perception of low-level nor high-level stimulus features, (2) a low-level group that consciously perceives the constituent line pattern, without exhibiting a high-level conscious representation, and (3) a high-level group that additionally exhibits a conscious high-level representation of the stimulus lines as a face. This was embedded into a novel IB paradigm, developed based on extended pilot studies, to render the critical stimulus irrelevant to the task but perceivable according to the assigned experimental group despite identical physical stimulation. Critically, this enabled us to investigate the relationship between ERPs and both a low- and high-level consciousness contrast (aware versus unaware conditions) under minimized task- and report-related processes.

The paradigm was designed to encompass three key characteristics of these experimental conditions. First, participants in the unaware group should not exhibit any conscious perception of the stimulus, distinguishing it from the original gorilla experiment^100^ and earlier IB studies focusing on high-level phenomena such as face-specific NCCs.^17^^,^^22^^,^^63^ In prior studies, “unaware” participants likely consciously perceived background elements constituting the critical stimulus, such as lines or a moving person dressed in black, just not engaging in further processing allowing them to identify the lines as a particular geometric form,^20^^,^^68^ face,^17^^,^^22^^,^^63^ letters^101^^,^^102^ or the moving black stimulus as a gorilla.^100^ The unconscious condition in previous studies, therefore, included neural activity related to low-level conscious perception. In contrast, the present study aimed to induce a genuine unconscious condition of the critical stimulus despite a sudden stimulus onset, sufficient stimulus strength, and a sufficient number of stimulus presentations. Second, low-level perception of the stimulus in the respective group should not rely on decreased visual clarity compared to the high-level perception, disentangling the manipulation of processing levels from visual clarity.^35^ Thus, participants in the low-level group should not exhibit a high-level perception of the critical stimulus at any point during the experiment, despite sufficient stimulus strength, a sufficient number of stimulus presentations, and high perceptual distinctiveness and conceptual salience of the stimulus. Third, face identification in the high-level group should involve complex feature integration, enabling the investigation of NCCs related to unequivocal high-level conscious representations under minimized task requirements.

To fulfill these experimental aims, we employed a complex face stimulus consisting of short lines (similarly introduced in a previous ERP study,^63^) embedded in a novel IB paradigm. The facial stimulus was derived from a single-line drawing of a human face, oriented obliquely upward to the right. The original line drawing was segmented into short, straight segments to match the experiment’s background. Based on extended pilot studies, this face stimulus was designed to leverage visual one-shot learning, enabling rapid and lasting recognition upon initial exposure to a less ambiguous version of the image (see Figure 1B).^103^ In the following, we will refer to the stimulus as a face stimulus to facilitate comprehension, although note that the stimulus was specified solely by lines instead of an actual face for the low-level conscious group.Visual stimuli were presented on an iiyama HM903DT monitor at 60 Hz with a resolution of 1920 × 1080 pixels and the experiment was implemented in MATLAB (Version R2019b; Mathworks Inc., Natick, MA; https://www.mathworks.com) and the Psychophysics Toolbox.^97^ All RGB values are reported in normalized format (0–1) as used in MATLAB. The stimuli appeared within a centrally positioned gray frame (680 × 680 px; RGB 0.4/0.4/0.4) against a lighter gray background (RGB 0.8/0.8/0.8). A green fixation cross (17 × 17 px, RGB 0/1/0; line width of 5 px) was presented at the screen’s center. Each trial consisted of task-irrelevant black line configurations (RGB 0/0/0) presented for 1000 ms. These line configurations were positioned based on an invisible 16 × 16 grid, dividing the central frame into 256 cells.

The experiment comprised three trial types (random, face, and catch trials; Figures 1A and 1D). In random trials, 40% of the grid cells (102) were randomly selected to contain a black line with random orientation (continuous angles between 0 and 360°), length (25 ± 20 px), and jittered position (cell center ±8 px horizontally and vertically) with an uniform line width of 2 px, producing a rhythmically changing, visually noisy background. Face trials followed the same configuration rules but displayed only 70 black lines, leaving 23 specific grid cells empty to ensure visibility of the critical stimulus. The face stimulus comprised 32 lighter lines (RGB 0.45/0.45/0.45, with alpha = 0.75; parameters optimized through pilot testing) and appeared either 300 or 600 ms after trial onset for a duration of 300 ms to ensure temporal distinctiveness. This onset timing was counterbalanced and randomized within blocks. After the face stimulus offset, the 70 black lines persisted for the remaining trial duration (400 or 100 ms, respectively). Catch trials were identical to face trials but omitted the face stimulus, serving both as a baseline control and to prevent stimulus anticipation. The denser background in random trials (102 lines) ensured continuous distraction, whereas the reduced density in face and catch trials preserved visibility of the critical stimulus in face trials. This configuration maximized distraction while enabling a controlled comparison between face and catch trials.

The main experiment consisted of 1500 trials divided into 20 blocks of 75 trials each. Each block began with 15 and ended with 10 random trials. The remaining 50 trials contained 5 face trials and 5 catch trials in randomized order, with a minimum of three trials between successive face trials. Thus, all participants, regardless of their experimental condition, were presented with exactly 100 face trials and 100 catch trials.

All participants engaged in a foreground visual distractor task, while task-irrelevant line configurations appeared in the background. The task involved a total of 26 moving rectangles displayed within a 600 × 600 px area centered on the fixation cross: 16 white rectangles (all 42 × 28 px; RGB 1/1/1) and 10 black rectangles (RGB 0/0/0), of which five were larger (42 × 28 px), and five were smaller (30 × 16 px). These rectangles moved at a constant speed of 3 pixels per frame, with trajectories updated upon same-color collisions following the law of reflection. To reduce variability between experimental groups and minimize calculation effort, we pre-generated 16 versions of rectangle movement patterns that were counterbalanced between groups. Each block began with a 5-s orientation period, during which rectangles were initially displayed with 10 × 3 px before transitioning to their experimental dimensions. All participants’ responses were entered using a standard keyboard. A chin rest was used to prevent head movements during the experiment, allowing for a standardized viewing distance of 60 cm.

#### Procedure

Prior to the experiment, participants were pseudorandomly assigned to one of three experimental groups that differed only in their prior knowledge of the critical stimulus (Figure 1C): an uninformed group received no information about the face stimulus, a low-level group was informed about additional bright lines appearing in the background, and a high-level group was informed that these lines constitute a face. The stimulus presentation was identical for all participants during both the main experiment and a preceding practice session. All participants were instructed to maintain fixation on the fixation cross while counting each time a large black rectangle crossed an imaginary horizontal midline through the fixation cross. At the beginning of each new block, they were asked to start from zero. In addition to the task, all participants were informed about the regular presentation of black lines in the background for additional distraction, which were task-irrelevant and instructed to be ignored. This task directed participants’ attention toward black, large-scale elements while requiring the suppression of white and smaller shapes (including white rectangles and lines) to perform the task effectively. This design enabled a repeated presentation of lighter lines with abrupt onsets in the noisy background that remained nonconscious to uninformed participants but conscious as well as perceptually and temporally distinct to informed participants. At the same time, the design provided sufficient stimulus intensity to enable higher-level perceptual processing in the respective high-level group.

During the information phase of the high-level group, participants viewed the face stimulus for three 2000-ms presentations, with a less ambiguous face drawing initially overlaid that gradually faded during each presentation to demonstrate how the facial features are embedded in the lines. Participants were then asked whether they had seen the face in the stimulus. Both the low- and high-level groups underwent identical information procedures thereafter, except for the low-level group viewing a modified stimulus (lines rearranged to prevent face perception) and confirming line detection, while the high-level groups confirmed face perception in respective prompts. The stimulus then appeared among 70 black lines for three 2000-ms presentations, with surrounding black lines gradually fading during each presentation. After confirming detection, participants viewed six alternating catch and face trials, where face trials contained a higher-contrast face stimulus (RGB 0.45/0.45/0.45; alpha: 1), appearing 400 ms post-trial-onset. After confirming detection again, participants viewed another six alternating trials (3 catch trials, 3 face trials), with face trials containing the stimulus at the contrast used during the experiment (alpha: 0.75), followed by a final confirmation. Non-detection at any phase triggered a restart of the instruction phase, with three restarts permitted before termination. No participant failed.

Prior to the experiment, participants of both informed groups were instructed that the stimulus was irrelevant to the task, and they were directed to focus solely on the large black rectangles. All participants completed a practice session of 20 random trials with reduced difficulty to familiarize themselves with the task. The practice trials featured only six black (three large) and six white rectangles moving at a slower speed of 2 px per frame. Participants were instructed to maintain steady fixation throughout the blocks to avoid eye movements and blinking, and to refrain from verbalizing their counts or making any movements. After each block, participants entered their crossing count using a standard keyboard. They received performance feedback indicating their accuracy on a six-point scale ranging from “this was very poor” to “this was excellent” (in German), determined by percentage deviation from the correct count. Feedback was nondirectional for deviations <40%, not indicating over- or undercounting, while deviations >40% received directional feedback (“much too low”/“much too high”). A mandatory break of at least 10 s followed each block to minimize eye strain and maintain attention. Participants initiated the next block at their discretion. Each block lasted 78 s, resulting in a total experiment duration of approximately 30 min.

Immediately after the experiment, participants completed an unannounced awareness assessment designed to identify those who detected the critical stimuli in the background and categorize their level of conscious perception. Figure 2A presents the distribution of perceptual responses to the critical face stimulus among all collected participants within each experimental condition prior to any exclusions for final analysis. The assessment started with an open question about any noticed background elements apart from the black lines, followed by a specific query about any bright lines in the background. Participants who reported having seen the light lines were further asked whether they perceived any coherent figures within them. The low-level group comprised only participants who reported no perception of figural content and were assigned to the low-level condition, thereby ensuring the absence of high-level perceptual representations concerning the critical stimulus. Aware participants were additionally asked for their subjective clarity of perception of the face stimulus on a scale from 1 to 10 (1 = extremely unclear, 10 = extremely clear), and to estimate the number of face stimuli (or lines respectively) they had perceived throughout the experiment to identify participants reporting very low exposure rates (<5 perceived stimuli; see Figure 2A Stimulus Ratings).

#### Experimental design and awareness contrasts

The current study implements a hierarchical design with three distinct awareness contrasts to investigate the neural correlates of visual consciousness across multiple representational levels. Comparing the low-level aware versus unaware group isolates neural activity associated with conscious perception of the stimulus’ elementary features (luminance-defined lines) in the absence of high-level face perception. The contrast between the high-level aware and unaware groups reflects the neural correlates of both conscious elementary feature perception and conscious integrated face perception. The high-level versus low-level contrast specifically isolates neural activity associated with conscious face perception while controlling for conscious perception of elementary visual features. This systematic design allows us to delineate the neural correlates of visual consciousness across multiple levels, from basic features to abstract integrated categorical face representation.

#### EEG data recording

A 64-channel BioSemi active electrode system (BioSemi B.V., Amsterdam, Netherlands) was employed to collect electrophysiological data. Electrodes were placed using the equiradial system conforming with BioSemi electrode caps. Furthermore, vertical and horizontal eye movements were recorded with two electrodes attached above and below the left eye and two electrodes attached to the right and left outer canthi. Instead of ground and reference, the BioSemi EEG system uses a CMS/DRL feedback loop with two additional electrodes. Electrical potentials were recorded with a sampling rate of 512 Hz, and electrode offsets were held below 20 μV. A built-in analog anti-aliasing low-pass filter of 104 Hz was applied prior to digitization.

#### EEG data preprocessing

The EEG data were preprocessed using Brain Electrical Source Analysis (BESA) Research 6.0 (BESA GmbH, Gräfeling, Germany). Offline, the data were re-referenced to average reference and filtered with a high-pass forward filter of 0.2 (6 dB/oct) and a 30 Hz low-pass zero-phase filter (24 dB/oct). Line noise was removed by applying a 50 Hz notch filter. Eye movements were corrected using the automatic eye-artifact correction method implemented in BESA.^104^ Further artifacts were removed using the semi-automatic, PCA-based artifact topography algorithm implemented in BESA by manually selecting artifacts. All corresponding components were removed automatically if they explained more than 80% variance of the artifact topography. The continuous signal was segmented into epochs of 500 ms before to 1500 ms after the onset of the critical background stimuli. Trials were baseline-corrected based on the average of the prestimulus interval from −200 to 0 ms. Segments with extreme amplitude thresholds defined individually by visual inspection were removed and bad channels were interpolated. In the EEG data of the final sample, an average of 1.95% trials (*SD* = 3.31%) were excluded from statistical analysis (no group differences: *F*(2, 82) = 0.12, *p* = 0.888), leading to an average of 97.84 (*SD* = 3.56) included face trials and 97.91 (*SD* = 3.79) catch trials. Furthermore, an average of 2.8 electrodes (*SD* = 1.98) were interpolated (no group differences: *F*(2, 82) = 0.83, *p* = 0.439).

### Quantification and statistical analysis

#### Behavioral data analysis

##### Task performance data analysis

Individual task performance was quantified using the normalized absolute error between counts and crossings (Deviation = |Counts - Crossings|/Crossings) calculated for each experimental block, with the median taken as the individual task performance measure to provide robustness against skewness and intraindividual outliers. Outliers in median deviation were identified as values exceeding 3 times the group-wise interquartile range and excluded from further analysis, to ensure comparable EEG data based on adequate attentional allocation and thorough understanding of the task.

Median deviations were compared between groups using one-way Welch’s analysis of variance (ANOVA), followed by two-tailed Games-Howell post hoc tests^105^^,^^106^ thereby accounting for potential variance inequality in combination with unequal sample sizes. We adopted a conventional significance threshold of α < 0.05 and report Holm-adjusted *p*-values along with Cohen’s d and partial ω^2^ as effect size measures.

In addition to traditional frequentist statistics, Bayesian analyses were conducted using the R package BayesFactor^107^ with default priors. The BF10 value quantifies how much more likely the alternative hypothesis is compared to the null hypothesis, while BF01 indicates the likelihood of the null hypothesis compared to the alternative hypothesis. In the Bayesian ANOVA, the factor version was included as a random effect (referring to the 16 versions of pre-generated rectangle movement patterns counterbalanced between groups). The reported BFM compares the model that includes both the fixed effect of the group and the random effect of the version against the null model, including only the random effect, indicating how much more likely the former model is.

##### Awareness rating analysis

Post-experimental delayed awareness ratings confirmed the effectiveness of the experimental manipulation. Only participants whose reported perception matched their assigned condition were included in the final analysis. Subjective clarity ratings of the critical stimulus were compared between low- and high-level groups using an independent-samples *t* test with Welch correction to account for unequal variances in combination with different sample sizes. Consistent with established IB studies,^17^^,^^21^^,^^22^ retrospective frequency estimates served solely as an exclusion criterion (<5 perceived stimuli) for increasing statistical power of EEG analysis. Yet they were not subjected to further analysis, given their heterogeneous reporting format (blockwise sums, total estimates, or ranges) and unreliability of unannounced estimates for unattended repetitive stimuli for finer differentiation beyond their intended screening purpose. For descriptive purposes, participants’ frequency estimates were converted to total estimation counts after summation, using upper bound values (e.g., 30 for ‘25–30 stimuli seen’), displayed in Figure 2A.

#### EEG data analysis

For EEG data analysis using the FieldTrip Toolbox in MATLAB,^96^ a robust cluster-based permutation testing approach was used, effectively addressing the multidimensional nature of EEG data while maintaining statistical power to detect meaningful effects across varied spatial and temporal scales. Channels and time intervals of interest were broadly predefined to increase statistical power and address the specific components of interest. This approach was theoretically guided by current debates on NCCs, particularly examining the relationship between different levels of conscious perception and their neural signatures - specifically early posterior and later, more anterior components.

Early negativities were examined at posterior electrodes (occipitotemporal and posterior parietal: Iz, O2, O1, Oz, PO7, P7, P9, PO3, P5, TP7, T7, PO8, P8, P10, PO4, POz, P6, TP8, T8), including time points between 100 and 350 ms after stimulus onset. Late positivities were tested in a broad frontocentroparietal selection of electrodes (i.e., F5, F3, F1, Fz, F2, F4, F6, FC5, FC3, FC1, FCz, FC2, FC4, FC6, C5, C3, C1, Cz, C2, C4, C6, CP5, CP3, CP1, CPz, CP2, CP4, CP6, P5, P3, P1, Pz, P2, P4, P6, PO3, POz, PO4) in a later time window between 350 and 600 ms after stimulus onset.^2^^,^^108^ This comprehensive electrode selection strategy enables a systematic investigation of different spatiotemporal dynamics of electrophysiological NCCs across levels of perceptual representation while addressing current debates about conscious perception.

Individual differential responses to face versus catch trials were computed within each awareness group (unaware, low-level, high-level). Student *t*-values for independent samples at each preselected spatiotemporal point were then calculated separately for all three awareness contrasts (low-level vs. unaware, high-level vs. unaware, high-level vs. low-level). To further analyze the EEG data, we employed the threshold-free cluster enhancement (TFCE)^98^^,^^99^ approach using the FieldTrip Toolbox in MATLAB,^96^ addressing the multidimensional spatiotemporal nature of EEG recordings and complexity of neural processes under investigation. Traditional methods for EEG analysis often reduce information by averaging predefined samples and electrodes, potentially missing effects that fall outside predefined spatiotemporal patterns due to, e.g., the complex paradigms, stimulus variations, or insufficient justification for data reduction. In contrast, the present TFCE accounts for correlation between neighboring channels and time points while strictly controlling the family-wise error rate via the permutation procedure described below. Specifically, the resulting *t*-values were transformed into TFCE scores. These scores quantify cluster-like support by integrating both the signal intensity (height: H) and information from neighboring samples (cluster extent: E) using the weighing parameters H = 2 and E = 1.^98^^,^^99^ Unlike other cluster-based techniques, this method does not require an arbitrary threshold for cluster formation and allows detection of both localized, high-amplitude and distributed, lower-amplitude effects in our data.^98^^,^^99^ The resulting TFCE scores for every time point retain local minima and maxima across the data. Intuitively, TFCE thereby scores each sample according to both its own strength and the extent of the surrounding activity that supports it, so that focal, strong effects and broader, weaker but coherent effects can both be detected without committing to an arbitrary cluster-forming threshold. Statistical significance and corresponding *p*-values were determined by comparing observed TFCE values against a null distribution created through 5000 random permutations, with significance threshold set at *α* < 0.05. To increase statistical sensitivity for components of interest, we performed one-sided tests based on the direction of effects: negative for early components (100–350 ms) in posterior electrodes, and positive for later components in frontocentroparietal (350–600 ms) electrodes. The use of one-sided tests was conceptually motivated by the aim to test literature-defined candidate components with characteristic polarity, electrodes, and latencies. Specifically, these t-tests were used across the three awareness contrasts to determine at which representational level of conscious perception each component emerges. Thus, directional hypotheses derived from prior literature were combined with an analysis approach that retained sensitivity to spatiotemporal variation in the observed effects.

##### Exploratory analyses

Additionally, we conducted two exploratory analyses. First, to investigate central awareness-related negativities recently reported in both visual^16^^,^^18^ and auditory^65^^,^^66^ modalities when controlling for post-perceptual confounds, we performed one-sided t-tests with TFCE correction for each of the three awareness contrasts (low-level vs. unaware, high-level vs. unaware, high-level vs. low-level), testing for negative differences in the predefined frontocentroparietal electrodes between 200 and 600 ms post-stimulus. Second, we examined specific P3b-related effects using a one-way ANOVA to compare differential ERP amplitudes (face-catch) at electrode Pz during the typical P3b time window (350–600 ms),^17^^,^^67^ complementing our primary analysis of late positive effects with a more conventional assessment. Further exploratory analyses without constraints regarding electrodes, polarity or time window are provided in Supplementary Materials.

##### Moderation analyses

Given the differential *a priori* information across awareness conditions regarding the visual stimulus, variations in attentional allocation that manifest in task performance may emerge despite uniform instructions to ignore the background^27^ and potentially confound genuine awareness-specific differences. To disentangle awareness-related effects from potential attention-driven confounds, we implemented moderation analyses for each cluster identified through our primary analysis procedure. For each cluster, mean amplitudes were extracted by averaging across significant samples of the cluster electrodes for individual participants. The hierarchical linear regression model was specified as:

$$Y=\beta_{0}+\beta_{1}\;\text{Group}1+\beta_{2}\;\text{Group}2+\beta_{3}\;\text{Performance}\;\text{centered}+\beta_{4}\;\text{Group}1\times \text{Performance}\;\text{centered}+\beta_{5}\;\text{Group}2\times \text{Performance}\;\text{centered}+\varepsilon$$

where *Y* represents the respective individual mean cluster amplitudes, *Group1* and *Group2* are dummy-coded group variables with the third group as reference according to the group contrast (e.g., unaware group as reference for cluster of high-level vs. unaware contrast), and *Performance centered* represents mean-centered absolute percent deviations. The moderation analyses examined whether the found clusters are influenced by task performance levels. Specifically, significant interaction terms would indicate that group differences in neural activity vary as a function of task performance, suggesting that performance differences (potentially reflecting attentional variations) contribute to the observed neural effects. Conversely, significant main effects of group with non-significant interaction terms would indicate that neural differences persist across performance levels, providing evidence that the observed effects are not primarily driven by performance-related confounds and may therefore reflect genuine awareness-specific processes. Moderation analyses were performed using the lm() function in R version 4.3.2.^109^
