# Supplementary material for: Distinct electrophysiological correlates of low- and high-level visual consciousness with minimized post-perceptual processing
Source: iScience. 2026 Oct 15;29(11):117853. doi: 10.1016/j.isci.2026.117853 (PMC13691566; doi:10.1016/j.isci.2026.117853)
Supplement: Supplementary file 1 [file mmc1.pdf]

iScience, Volume *29*

Supplemental information

Distinct electrophysiological correlates of low- and high-level visual consciousness with minimized post-perceptual processing

Annika Hense, Antje Peters, Torge Dellert, Maximilian Bruchmann, and Thomas Straube

Supplement

Exploratory analysis

In addition to the predefined hypothesis-driven analysis in the main text, we also present a completely exploratory analysis for further inspection of the data. Please note that significant effects in the same time windows are not independent with, for example, positivities in frontal regions in the VAN time window due to the average reference.

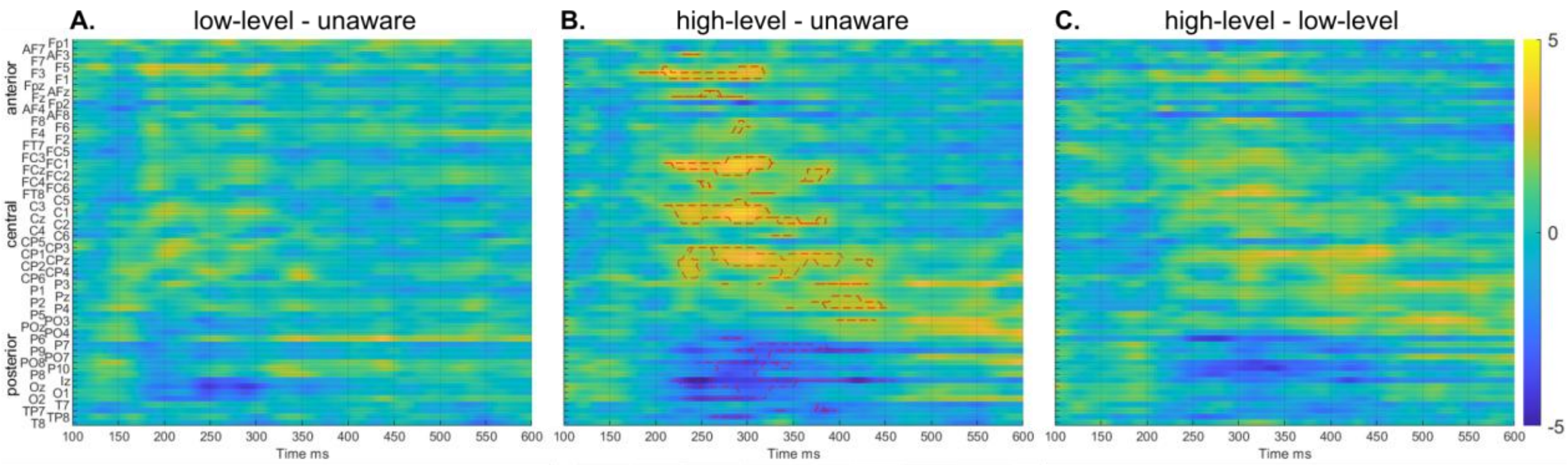

Figure S1: Results from the mass univariate analysis of EEG data for all three awareness without a-priori channel selection and using an interval of interest of 100-600 ms. The maps show significant differences in stimulus-specific responses (face - catch trials) for each awareness contrast. Each row on the y-axis represents an individual electrode, with time plotted in milliseconds on the x-axis from 100-600 ms post-stimulus onset. Significant t-values (p < 0.05) after TFCE correction are surrounded by dashed lines

Moderation analysis

Table S1

Results of Moderation Analysis for Significant Clusters in Low-level vs. Unaware Contrast

**Predictor**	**Low-level vs. Unaware**			
	negative			
	**b ± SE**	***t***	***p***	
Intercept	0.40 ± 0.18	2.220	0.029	*
High-level (vs. Ref.)	-0.83 ± 0.25	-3.287	0.002	**
**Low-level (vs. Ref.)**	**-0.74 ± 0.26**	**-2.833**	**0.006**	**
Unaware (vs. Ref.)	-	-	-	
Performance (centered)	-7.28 ± 4.60	-1.580	0.118	
High-level x Performance	6.77 ± 5.52	1.226	0.224	

**Low-level x Performance**	**5.75 ± 6.54**	**0.879**	**0.382**	
Unaware x Performance	-	-	-	
**Adj. R^2**	0.177			
**F(5,79)**	4.601		0.001	**

Note.**p* < .05, ***p* < .01, ****p < .001*

Table S2

Results of Moderation Analysis for Significant Clusters in High-level vs. Unaware Contrast

**Predictor**	**High-level vs. Unaware** negative			
	**b ± SE**	***t***	***p***	
Intercept	0.43 ± 0.13	3.303	0.001	**
**High-level (vs. Unaware)**	**-0.78 ± 0.18**	**-4.233**	**<0.001**	***
Low-level (vs. Unaware)	-0.24 ± 0.19	-1.241	0.218	
-	-	-	-	
Performance (centered)	-2.85 ± 3.35	-0.850	0.218	
**High-level x Performance**	**2.70 ± 4.02**	**0.671**	**0.504**	
Low-level x Performance	2.96 ± 4.76	0.623	0.535	
-	-	-	-	
**Adj. R^2**	0.196			
**F(5,79)**	5.107		<0.001	***

**Predictor**	**High-level vs. Unaware** Positive 1			
	**b ± SE**	***t***	***p***	
Intercept	-0.01 ± 0.10	-0.051	0.959	
**High-level (vs. Unaware)**	**0.56 ± 0.14**	**3.904**	**<0.001**	***
Low-level (vs. Unaware)	0.11 ± 0.15	0.758	0.450	
-	-	-	-	
Performance (centered)	-1.59 ± 2.62	-0.608	0.545	
**High-level x Performance**	**0.87 ± 3.14**	**0.278**	**0.782**	
Low-level x Performance	2.26 ± 3.72	0.607	0.545	

-	-	-	-	
**Adj. R^2**	0.134			
**F(5,79)**	3.588		0.006	**

**Predictor**	**High-level vs. Unaware**<br>Positive 2			
	**b ± SE**	***t***	***p***	
Intercept	-0.02 ± 0.13	-0.126	0.900	
**High-level (vs. Unaware)**	**0.74 ± 0.18**	**4.093**	**<0.001**	***
Low-level (vs. Unaware)	0.19 ± 0.19	1.032	0.305	
-	-	-	-	
Performance (centered)	-3.92 ± 3.29	-1.194	0.236	
**High-level x Performance**	**3.43 ± 3.94**	**1.124**	**0.264**	
Low-level x Performance	7.74 ± 4.66	1.659	0.101	
-	-	-	-	
**Adj. R^2**	0.169			
**F(5,79)**	4.406		0.001	**

Note.*$p < .05$, **$p < .01$, ***$p < .001$

Table S3

Results of Moderation Analysis for Significant Clusters in High-level vs. Low-level Contrast

**Predictor**	**High-level vs. Low-level**<br>Negative			
	**b ± SE**	***t***	***p***	
Intercept	0.69 ± 0.15	4.733	<0.001	***
**High-level (vs. Low-level)**	**-0.96 ± 0.20**	**-4.792**	**<0.001**	***
Unaware (vs. Low-level)	-0.19 ± 0.20	-0.936	0.352	
-	-	-	-	
Performance (centered)	1.01 ± 3.54	0.286	0.775	
**High-level x Performance**	**0.70 ± 4.23**	**0.166**	**0.868**	
Unaware x Performance	-0.77 ± 4.99	-0.154	0.878	
-	-	-	-	
**Adj. R^2**	0.214			
**F(5,79)**	5.575		<0.001	***

**Predictor**	**High-level vs. Low-level**<br>Positive 1			
	**b ± SE**	***t***	***p***	
Intercept	0.04 ± 0.11	0.334	0.739	
**High-level (vs. Low-level)**	**0.56 ± 0.15**	**3.797**	**<0.001**	***
Unaware (vs. Low-level)	0.12 ± 0.15	0.829	0.409	
-	-	-	-	
Performance (centered)	1.27 ± 2.63	0.483	0.630	
**High-level x Performance**	**-2.85 ± 3.14**	**-0.906**	**0.368**	
Unaware x Performance	-4.99 ± 3.70	-1.348	0.181	
-	-	-	-	
**Adj. R^2**	0.136			
**F(5,79)**	3.634		0.005	**

**Predictor**	**High-level vs. Low-level** Positive 2			
	**b ± SE**	***t***	***p***	
Intercept	0.15 ± 0.16	0.912	0.365	
**High-level (vs. Low-level)**	**0.68 ± 0.23**	**3.008**	**0.004**	**
Unaware (vs. Low-level)	0.02 ± 0.22	1.670	0.099	
-	-	-	-	
Performance (centered)	6.66 ± 3.99	1.670	0.099	
**High-level x Performance**	**-4.88 ± 4.77**	**-1.022**	**0.310**	
Unaware x Performance	-7.73 ± 5.62	-1.376	0.173	
-	-	-	-	
**Adj. R^2**	0.149			
**F(5,79)**	3.932		0.003	**

Note.*$p < .05$, **$p < .01$, ***$p < .001$

Scatter plots for correlations between task performance and ERP amplitudes in the high-awareness group

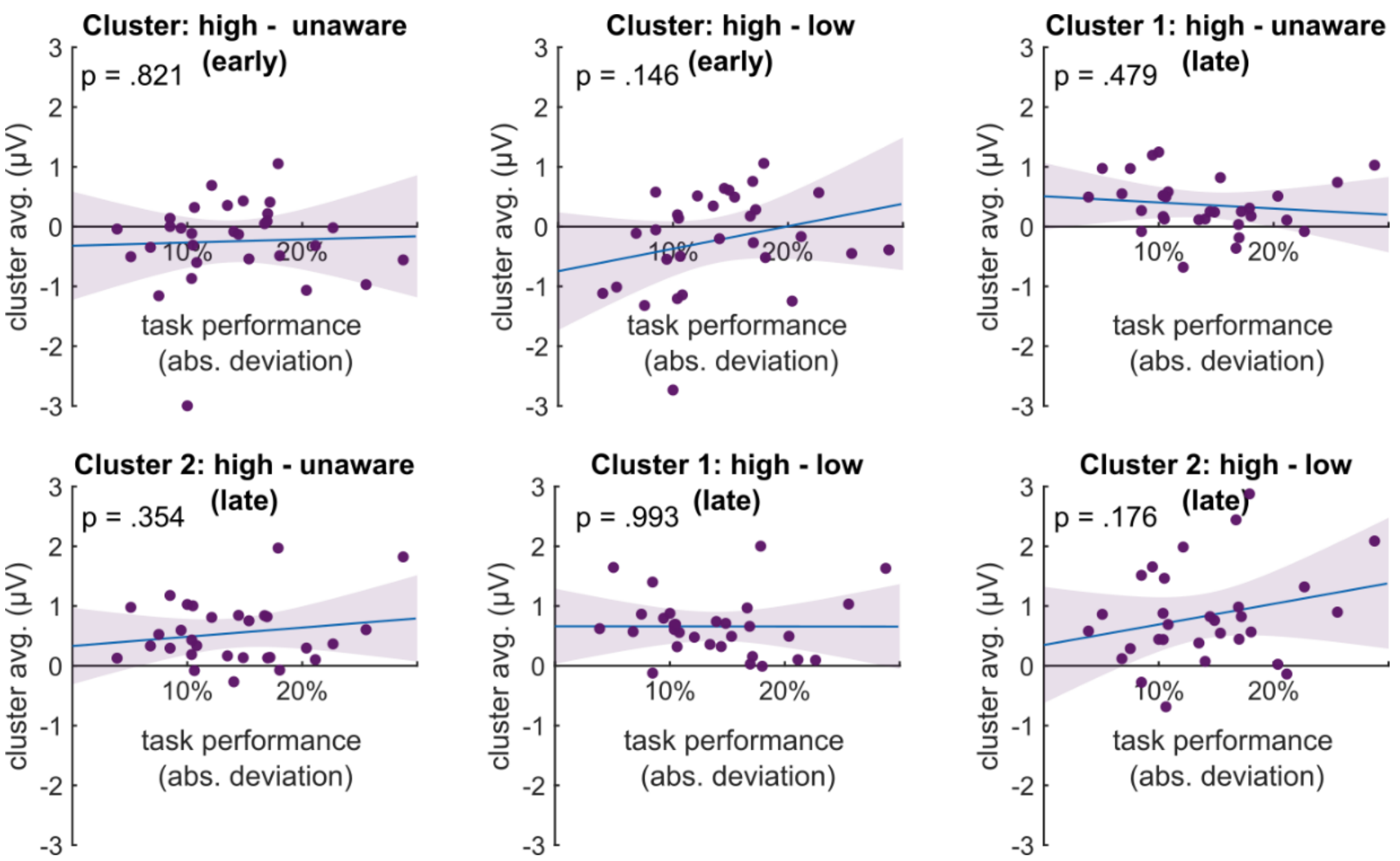

Figure S2: Scatter plots illustrating the correlation between task performance of the high-awareness group (x-axis) and ERP amplitudes (y-axis) observed at the six significant clusters, two in the early interval (see main text Figure 4) and four in the late interval (see main text Figure 5), that involved the high-awareness group. Each plot contains a linear regression line

and the shaded areas represent 95% confidence bands. The depicted p-values refer to the significance test of the corresponding Pearson correlation coefficient.
